# Prevalence and associated factors of delayed sputum smear conversion in patients treated for smear positive pulmonary tuberculosis: A retrospective follow up study in Sabah, Malaysia

**DOI:** 10.1371/journal.pone.0282733

**Published:** 2023-03-06

**Authors:** Linghui Amanda Khor, Ulfa Nur Izzati A. Wahid, Lee Lee Ling, Sarah Michael S. Liansim, Jush’n Oon, Mahendran Naidu Balakrishnan, Wei Leik Ng, Ai Theng Cheong

**Affiliations:** 1 Luyang Health Clinic, Kota Kinabalu, Sabah, Malaysia; 2 Tamparuli Health Clinic, Tuaran, Sabah, Malaysia; 3 Penampang Health Clinic, Penampang, Sabah, Malaysia; 4 Permai Polyclinic Sri Kepayan, Kota Kinabalu, Sabah, Malaysia; 5 Department of Primary Care Medicine, Faculty of Medicine, Universiti Malaya, Kuala Lumpur, Malaysia; 6 Department of Family Medicine, Faculty of Medicine and Health Sciences, Universiti Putra Malaysia, Serdang, Selangor, Malaysia; The University of Georgia, UNITED STATES

## Abstract

**Introduction:**

Tuberculosis remains a major health problem globally and in Malaysia, particularly in the state of Sabah. Delayed sputum conversion is associated with treatment failure, drug-resistant tuberculosis and mortality. We aimed to determine the prevalence of delayed sputum conversion among smear positive pulmonary tuberculosis (PTB) patients and its associated factors in Sabah, Malaysia.

**Methods:**

A retrospective follow up study on all patients newly diagnosed with smear positive pulmonary tuberculosis from 2017 to 2019 was conducted at three government health clinics in Sabah, utilizing data from a national electronic tuberculosis database and medical records. Descriptive statistics and binary logistic regression were applied for data analysis. The outcome of the study was the sputum conversion status at the end of the two-month intensive treatment phase with either successful conversion to smear negative or non-conversion.

**Results:**

374 patients were included in the analysis. Our patients were generally younger than 60 years old with no medical illness and varying proportions of tuberculosis severity as judged by radiographic appearance and sputum bacillary load upon diagnosis. Foreigners constituted 27.8% of our sample. 8.8% (confidence interval: 6.2–12.2) did not convert to smear negative at the end of the intensive phase. Binary logistic regression showed that older patients ≥60 years old (adjusted odds ratio, AOR = 4.303), foreigners (AOR = 3.184) and patients with higher sputum bacillary load at diagnosis [2+ (AOR = 5.061) and 3+ (AOR = 4.992)] were more likely to have delayed sputum smear conversion.

**Conclusion:**

The prevalence of delayed sputum conversion in our study was considerably low at 8.8% with age ≥60 years old, foreigners and higher pre-treatment sputum bacillary load associated with delayed conversion. Healthcare providers should take note of these factors and ensure the patients receive proper follow up treatment.

## Introduction

Tuberculosis (TB) remains a significant health problem globally. It is estimated there were 1.6 million deaths due to tuberculosis worldwide in 2021, following an upward trend from 1.4 million in 2019 and 1.5 million in 2020 [1]. TB is expected to rank second only to COVID-19 as the cause of death from a single infectious agent in 2020 and 2021 [1]. The Southeast Asia region bears the highest TB burden. In 2021, 45% of new TB cases were reported in Southeast Asia, followed by Africa (23%), Western Pacific (18%) and smaller proportions in other regions [1].

The main source of transmission for TB is from smear positive pulmonary TB (PTB) patients via infective droplets from their lungs and throat [2]. Direct microscopic observation of sputum smear for acid-fast bacilli (AFB) plays an important role in treatment monitoring and this method is widely and easily available in developing countries such as Malaysia [3]. As recommended by World Health Organization (WHO), sputum conversion rate (SCR) to smear negative from a smear positive patient at the end of the intensive phase of anti-tuberculosis therapy (ATT) (at the end of the second month of treatment duration), is an operational indicator of the national TB control programs’ capacity and an essentially important clinical indicator of treatment response and disease prognosis [4]. Delayed sputum conversion, defined as non-conversion to smear negative PTB at the end of intensive phase, is associated with poorer outcomes, specifically treatment failure, and increased risk of drug resistance and higher mortality [5–7]. Delayed sputum conversion also contributes to higher treatment cost and additional burden to healthcare services, In Malaysia, treatment success rate for TB was 78% in 2020, lower than the global success rate of 86% in the same year [1]. Tackling delayed sputum conversion is one important strategy to improve the TB treatment success rate.

In Sabah, one of the states in Borneo Malaysia, TB notification constituted 20% of all TB notification nationwide between 2012 and 2018, despite representing only about 10% of the Malaysian population [8]. The TB incidence rate in Sabah during that period was reported as 128 per 100,000 population, which was higher than the national incidence rate (97 per 100,000 population) [9]. Sabah also has a unique and diverse sociodemographic composition compared to other states in Malaysia, comprising 42 ethnic groups, almost 200 sub-ethnic groups and a large proportion of immigrants, both legal and illegal, from neighbouring countries such as Philippines and Indonesia.

In this study, we aimed to determine the prevalence of delayed sputum conversion in patients with smear positive PTB and its associated factors on the west coast of Sabah. It is useful to obtain insight into the extent of delayed sputum conversion in this region with high TB burden and unique demographic profile. Understanding the factors associated with delayed sputum conversion could guide the development of public health policy to tackle this issue.

## Methods

### Study design and setting

A retrospective follow up study was conducted involving all new cases of patients with smear positive PTB at three health clinics on the central west coast of Sabah, namely Tamparuli Health Clinic in Tuaran, Penampang Health Clinic in Penampang and Luyang Health Clinic in Kota Kinabalu, from 1^st^ January 2017 to 31^st^ December 2019.

These three clinics were purposely selected because they were the main clinic that treat tuberculosis in three different districts in Sabah, namely Tuaran, Penampang and Kota Kinabalu. These three clinics acted as the main treating centre for tuberculosis in their respective district. Each TB unit in the respective clinic consisted of medical officer, medical assistant and nurses. All patients suspected of having PTB would be required to submit at least two sputum specimens for microscopic examination with at least one early morning specimen when possible. Sputum specimens were sent for acid-fast bacilli (AFB) smear and culture. AFB smears were performed routinely in the clinics using Ziehl-Nielsen stain and were examined under direct microscopy by medical laboratory technologists (MLT). MLT in health clinics are trained in analyzing the AFB smear and routinely receive refresher courses. Patients with smear positive PTB could be started on treatment in any of these clinics. Cases like smear negative PTB, extrapulmonary TB and drug-resistant TB such as multidrug-resistant TB (MDR-TB) would be referred to the hospital for treatment initiation. Once diagnosed and started on treatment, they could continue treatment in these clinics. Medication was provided on a daily basis for intensive phase and weekly basis for maintenance phase. All relevant information from case note was entered into MyTB system, an electronic TB clinical registry system operated by the Ministry of Health Malaysia.

### Study population

We included all patients newly diagnosed with smear positive PTB who were 18 years old and above, and under regular follow up at the study sites until the end of the two-month intensive phase. We included patients with drug-resistant TB as well. Patients who defaulted treatment before the end of the two-month intensive phase were excluded from the study because sputum conversion would only be monitored at the end of intensive phase. We also excluded those with disseminated tuberculosis because they were being followed up in hospitals instead of health clinics.

### Data collection

We used universal sampling and extracted all cases that fulfilled our study criteria from the MyTB database. Information that was readily available from MyTB database was the age at diagnosis, ethnicity, nationality, gender, education level, smoking status at diagnosis, diabetes and human immunodeficiency virus (HIV) status at diagnosis, sputum AFB load at diagnosis, chest X-ray (CXR) severity at diagnosis, presence of MDR-TB and status of sputum conversion at the end of the two-month intensive phase. Some information that was not available from the MyTB database such as presence of other co-morbidities, alcohol dependence status at diagnosis, duration of symptoms before diagnosis and number of days missing directly observed therapy (DOT) were obtained from the patients’ manual medical records that were kept in the respective health clinics. All data were recorded using data collection form.

### Variables and operational definition

Smear-positive PTB was diagnosed in either one of the following ways (10): (i) two or more positive sputum AFB smears, or (ii) one positive sputum AFB smear accompanied by abnormalities in CXR suggestive of PTB (as determined by the physician), or (iii) one positive sputum AFB smear and one positive sputum culture *Mycobacterium tuberculosis*.

The dependent variable was the delayed sputum conversion, defined as smear-positive PTB with a sputum sample that remains AFB smear-positive at the end of the two-month intensive phase of anti-tuberculous treatment.

The independent variables were the age at diagnosis, ethnicity, nationality, gender, education level, smoking status at diagnosis, alcohol dependence status at diagnosis, presence of diabetes mellitus at diagnosis, co-morbidities, HIV status at diagnosis, sputum AFB load at diagnosis, CXR severity at diagnosis, duration of symptoms before diagnosis, presence of MDR-TB and number of days missing DOT. Alcohol dependence was based on the fulfilment of three or more DSM-IV dependence criteria of substance use disorders within 12 months [10]. Presence of diabetes mellitus at diagnosis was defined as patients who were diagnosed with diabetes mellitus at the point of diagnosis of TB or were previously diagnosed by physician with two abnormal glucose results (if asymptomatic), one abnormal glucose result (if symptomatic), or HbA1c >6.3% based on Malaysian guideline [11]. Co-morbidities included medical conditions that were either recorded in the MyTB database or medical records in clinic, such as hypertension, dyslipidemia, ischaemic heart disease, hepatitis B, chronic kidney disease, stroke and peptic ulcer disease. HIV status at diagnosis referred either to patients with pre-existing HIV, or who were newly diagnosed with upon diagnosis of TB. Sputum AFB load was graded as scanty, 1+, 2+, and 3+ based on the number of AFB seen microscopically before initiation of treatment (negative: no bacilli per 100 fields of observation; scanty: 1–9 bacilli per 100 fields; 1+: 10 to 99 bacilli per 100 fields; 2+: 1–10 per field; and 3+: >10 per field of observation). CXR severity at diagnosis was graded by treating physicians into minimal, moderate or far advanced based on Malaysian guideline [12]. Duration of symptoms before diagnosis was defined by the duration of symptoms suggestive of PTB from the time of onset to the point of diagnosis, based on the history documented by physician in the medical records. MDR-TB was defined as strains of TB that are resistant to at least two main first-line antituberculous drugs (i.e. isoniazid and rifampicin), as demonstrated in the sputum culture and sensitivity result (10). Number of days missing DOT was defined as the number of days patient missed the antituberculous medication during the intensive phase as documented in the medical records.

To maintain the quality of data, validation rules were implemented in the Microsoft Excel sheet for data entry. Standardized vocabularies and units were used with regular sessions among researchers to discuss any irregularities. Completed data entries were screened to detect any irregularities.

### Data analysis

Data was entered and analyzed using IBM Statistical Program for Social Sciences (SPSS) software version 26. The categorical data were presented as frequency and percentage. For the age variable, 60 years old was chosen as the cut-off point for analysis because Malaysia public healthcare system defined people aged 60 years and above as older persons or senior citizens with healthcare policies for older persons designed around this cut-off point of age. The outcome of this study (dependent variable) was sputum conversion at the end of the 2-month intensive phase, with either successful conversion to smear negative or non-conversion (delayed sputum conversion). The association between the dependent and independent variables was examined by Chi-square test. The assumptions for Chi-square test were checked; all expected frequencies were greater than 1 and at least 80% are greater than 5. The level of significance was set at p-value of less than 0.05. Significant variables in Chi-square test and variables which might have clinical importance to predict delayed sputum conversion, based on literature review, researchers’ experiences and observations, were included in the bivariable logistic regression. Bivariable and multivariable binary logistic regression were performed to determine the association between the independent variables with delayed sputum conversion. Variables with p-value <0.25 from bivariable logistic regression were included in the multivariable logistic regression model. Literature supports the inclusion of p-value < 0.25 into the multivariable regression analysis [13]. Adjusted odds ratio was calculated with p-value of less than 0.05 considered statistically significant. Model fit was checked with Hosmer and Lemeshow test and classification table.

### Ethics

Ethical approval for this study was obtained from the Medical Research & Ethics Committee, Ministry of Health Malaysia (NMRR-20-1581-53331). The data extracted from the MyTB database were de-identified to protect patients’ confidentiality. Patients’ consent was not required by the ethics committee as the study only analyzed secondary data from the database and medical records.

## Results

A total of 374 new cases of smear positive pulmonary tuberculosis that fulfilled the inclusion criteria were included in this study. The sociodemographic and clinical characteristics of the cohort were shown in Table 1. The majority of the patients were male in the age group of 18 to 59 years old. About one-third of our cohort (27.8%, n = 104) was foreigner.

**Table 1:** Sociodemographic and clinical characteristics of patients smear positive pulmonary tuberculosis (N = 374).

Sociodemographic characteristics	Frequency, n (%)
**Age** • 18 to 59 years old • 60 years old and above	319 (85.3%) 55 (14.7%)
**Gender** • Male • Female	250 (66.8%) 124 (33.2%)
**Nationality** • Malaysian • Foreigner	270 (72.2%) 104 (27.8%)
**Educational level** • Primary/ No formal education • Secondary/ Tertiary • Unknown	98 (26.2%) 114 (30.5%) 162 (43.3%)
**Clinical characteristics**	**Frequency, n (%)**
**Comorbidities** • No known medical illness • 1 comorbid • 2 comorbid and more	267 (71.4%) 70 (18.7%) 37 (9.9%)
**HIV status** • Non-reactive • Reactive	369 (98.7%) 5 (1.3%)
**Presence of diabetes mellitus** • Yes • No	43 (11.5%) 331 (88.5%)
**Smoking status** • Non-smoker • Active smoker • Ex-smoker	206 (55.1%) 105 (28.1%) 63 (16.8%)
**Alcohol dependence status** • No • Yes	301 (80.5%) 73 (19.5%)
**Severity of CXR** • Mild/ No lesion • Moderate • Severe	116 (31.0%) 165 (44.1%) 93 (24.9%)
**Duration of symptoms before diagnosis** • 1 month or less • 2 to 6 months • 7 months and above	206 (55.0%) 130 (34.8%) 38 (10.2%)
**Sputum AFB load at diagnosis** • Scanty • 1+ • 2+ • 3+	84 (22.5%) 139 (37.2%) 85 (22.7%) 66 (17.6%)
**Presence of missed DOT** • No • Yes	312 (83.4%) 62 (16.6%)
**Presence of MDR-TB** • No • Yes	370 (98.9%) 4 (1.1%)

The majority of our cohort had no known medical illness (71.4%, n = 267) with only 11.8% (n = 43) had diabetes mellitus and 1.3% (n = 5) had HIV. Most patients did not smoke (55.1%, n = 206) and consume alcohol (80.5%, n = 301). More patients had moderate CXR severity (44.1%, n = 165), 1+ AFB load at diagnosis (37.2%, n = 139) and symptoms for a month or less at diagnosis (55.0%, n = 206). Majority of the patients adhered to DOT (83.4%, n = 312) and only 1.1% (n = 4) were diagnosed with drug-resistant TB, which were all MDR-TB.

Out of these 374 patients with smear positive pulmonary tuberculosis, 33 (8.8%, 95% confidence interval, CI: 6.2–12.2) were non-converters at the end of the intensive phase which was at the second month of anti-tuberculous therapy, thus classified as having delayed sputum smear conversion.

For the bivariate analysis using Chi-square (Table 2) and simple logistic regression (Table 3), age group and sputum AFB load at diagnosis were significantly associated with delayed sputum smear conversion (p<0.05). Bivariate analysis and simple logistic regression were not performed for the presence of HIV and MDR-TB as the frequency in those variables were too small for meaningful analysis (5 or less).

**Table 2:** Chi-square test for association of sociodemographic and clinical variables with delayed sputum smear conversion.

Variables	Converted (N = 341)	Not Converted (N = 33)	P-value
**Age** <60 years old ≥60 years old	295(92.5%) 46 (83.6%)	24 (7.5%) 9 (16.4%)	0.033*
**Gender** Male Female	228 (91.2%) 113 (91.1%)	22 (8.8%) 11 (8.9%)	0.982
**Nationality** Malaysian Foreigner	250 (92.6%) 91 (87.5%)	20 (7.4%) 13 (12.5%)	0.120
**Educational level** Primary/ No formal education Secondary/ Tertiary Unknown	88 (89.8%) 101 (88.6%) 152 (93.8%)	10 (10.2%) 13 (11.4%) 10 (6.2%)	0.274
**Smoking status** Non-Smoker Active Smoker Ex-Smoker	186 (90.3%) 96 (91.4%) 59 (93.7%)	20 (9.7%) 9 (8.6%) 4 (6.3%)	0.709
**Comorbidities** No known medical illness 1 comorbid 2 comorbids or more	241 (90.3%) 65 (92.9%) 35 (94.6%)	26 (9.7%) 5 (7.1%) 2 (5.4%%)	0.588
**Presence of diabetes mellitus** Yes No	39 (90.7%) 302 (91.2%)	4 (9.3%) 2 (8.8%)	0.906
**Severity of CXR** No lesion/ Mild Moderate Severe	109 (94.0%) 152 (92.1%) 80 (86.0%)	7 (6.0%) 13 (7.9%) 13 (14.0%)	0.112
**Alcohol dependence status** Yes No	275 (91.4%) 66 (90.4%)	26 (8.6%) 7 (9.6%)	0.797
**Sputum AFB load** Scanty 1+ 2+ 3+	81 (96.4%) 135(97.1%) 71 (83.5%) 54 (81.8%)	3 (3.6%) 4 (2.9%) 14 (16.5%) 12 (18.2%)	0.000*
**Presence of missed DOTS** No Yes	285 (91.3%) 56 (90.3%)	27 (8.7%) 6 (9.7%)	0.795
**Duration of symptoms before diagnosis** One month or less 2 to 6 months 7 months and above	192 (93.2%) 113 (86.9%) 36 (94.7%)	14 (6.8%) 17 (13.1%) 2 (5.3%)	0.102

Item in asterisk (*) indicates p-value < 0.05.

**Table 3:** Bivariable and multivariable logistic regression for association of sociodemographic and clinical variables with delayed sputum smear conversion.

Variables	Crude OR	95% CI	P-value	AOR	95% CI	P-value
**Age** <60 years old ^ref^ ≥60 years old	2.405	1.052–5.497	0.037*	4.303	1.405–13.176	0.011*
**Gender** Male ^ref^ Female	1.009	0.473–2.153	0.982			
**Nationality** Malaysian ^ref^ Foreigner	1.786	0.853–3.737	0.124	3.184	1.184–8.563	0.022*
**Education level** Primary/ no formal education ^ref^ Secondary/Tertiary Unknown	1.133 0.579	0.473–2.710 0.232–1.445	0.780 0.242	2.481 0.694	0.795–7.737 0.238–2.023	0.117 0.504
**Comorbidities** Nil ^ref^ 1 comorbid 2 comorbid or more	0.713 0.530	0.262–1.930 0.120–2.330	0.505 0.400	0.450 0.379	0.136–1.493 0.071–2.018	0.192 0.256
**Presence of DM** No ^ref^ Yes	1.068	0.356–3.200	0.906			
**Smoking status** Non-smoker ^ref^ Active smoker Ex-smoker	0.872 0.631	0.382–1.988 0.207–1.918	0.744 0.417			
**Alcohol dependence status** No ^ref^ Yes	1.122	0.467–2.696	0.797			
**Severity of CXR** Mild/no lesion ^ref^ Moderate Severe	1.332 2.530	0.514–3.448 0.966–6.629	0.555 0.059	0.730 0.998	0.252–2.116 0.308–3.232	0.563 0.997
**Duration of symptoms before diagnosis** 1 month or less ^ref^ 2 to 6 months 7 months and above	2.063 0.762	0.980–4.344 0.166–3.497	0.057 0.727	1.685 0.590	0.740–3.839 0.118–2.954	0.214 0.521
**Sputum AFB load** Scanty ^ref^ 1+ 2+ 3+	0.800 5.324 6.000	0.175–3.665 1.470–19.283 1.617–22.263	0.774 0.011* 0.007*	0.665 5.061 4.992	0.138–3.197 1.315–19.478 1.249–19.947	0.665 0.018* 0.023*
**Presence of missed DOTs** No ^ref^ Yes	1.131	0.446–2.866	0.795			

OR = odds ratio, AOR = adjusted odds ratio, CI = confidence interval, Ref = reference. Items in asterisk (*) indicates p-value < 0.05. No multicollinearity was found; Hosmer and Lemeshow test, p-value = 0.735; The classification table is 91.2% correctly classified

Variables with significance of p<0.250 in univariate analysis were included in the multivariable logistic regression such as age, nationality, education level, severity of CXR, duration of symptoms and sputum AFB load. After controlling for all these factors, only age, nationality and sputum AFB load at diagnosis were found to be significantly associated with delayed sputum smear conversion. Older patients ≥60 years old (adjusted odds ratio, AOR = 4.303), foreigners (AOR = 3.184) and patients with higher sputum AFB load at diagnosis [2+ (AOR = 5.061) and 3+ (AOR = 4.992)] were more likely to have delayed sputum smear conversion. This model fit was based on the Hosmer and Lemeshow test which showed non-significant result (p-value = 0.735) and the percentage from the classification table (91.2% correctly classified).

## Discussion

Key findings from our study are: 1) the prevalence of delayed sputum conversion from 2017 to 2019 in our cohort is 8.8% (CI 6.2–12.2); 2) Older patients ≥ 60 years old, foreigners and patients with higher sputum AFB load at diagnosis are associated with delayed sputum conversion.

Table 4 outlines the comparison of our prevalence data with a few recent studies in the past five years. Our finding is consistent with another Malaysian study which was also conducted in Sabah in different research sites, where they identified 7.2% of patients having delayed sputum conversion from 2013 to 2018 [14]. In comparison, our prevalence of delayed sputum conversion was not too high. Ibrahim (2022) and Bhatti (2021) reported a higher prevalence of 19.1% and 30.5% respectively in Malaysia [15, 16]. Similar large variation of prevalence data was observed globally as observed in Table 4 (8.3% to 35%), and as reported in earlier studies, from 8% in Cameroon up to 30% in Cleveland [14–21]. This variation is observed despite the relatively standardized, effective first-line regime for antituberculous therapy worldwide. The different sociodemographic profiles may be one explanation. For example, Ibrahim (2022) focused on aboriginal groups in Peninsular Malaysia (separated from Borneo Malaysia) where the prevalence was much higher at 19.1% with no overlapping confidence limit with ours and Mokti’s (2021) study [14, 16]. Poor health accessibility may be a factor here in this group, leading to poor adherence and delayed sputum conversion. Likewise, health accessibility is an issue observed globally and is an important one to tackle as tuberculosis typically affects lesser developed countries [1].

**Table 4:** Comparison of prevalence of delayed sputum conversion and its associated factors in recent studies (year 2017–2022).

No	Author (Year)	Country	Prevalence, % (Confidence interval)	Associated factors
1	Our study	Sabah, Malaysia	8.8 (6.2–12.2)	Age≥60 Foreigner Higher sputum bacillary load
2	Ibrahim MN (2022)	Peninsular Malaysia (Aborigine group)	19.1 (15.7–22.9)	Smoking Diabetes mellitus HIV infection
3	Mokti K (2021)	Sabah, Malaysia	7.2 (6.2–8.2)	Moderate to advanced CXR Age> 60 Smoking No DOTS supervisor Non-Malaysian Suburban residence
4	Gunda (2017)	Rural Tanzania	8.3 (4.5–13.8)	Male Age>50 Sputum bacillary load of 3+
5	Azza (2019)	Tunisia	35 (31–40)	Diabetes mellitus Smoking Haemoptysis Higher sputum bacillary load
6	Bhatti (2021)	Penang, Malaysia (hospital patients)	30.5 (27.8–33.3)	Age≥50 Blue-collar jobs Smoking Higher sputum bacillary load Relapse and interruption in treatment
7	Asemahagn (2021)	Ethiopia	15.0 (11.0–19.6)	Higher BMI Higher sputum bacillary load HIV infection Diabetes mellitus Smoking Societal stigma, delay in TB service

We found that the following variables were significantly associated with delayed sputum conversion in our cohort: age ≥ 60 years old, foreigner status and higher sputum AFB load at diagnosis. Older age and higher sputum AFB load have consistently been shown to be associated with delayed conversion in previous studies [2, 14, 15, 17, 18]. Older age can lead to poorer immune response, causing ineffective clearance of the bacilli. Delay in timely health-seeking behaviour was also observed in older person, leading to poor progress in treatment for tuberculosis where regular, close follow up in intensive phase is important [22]. The higher sputum AFB load at diagnosis an indication of a heavier mycobacterial burden. More time may be needed to clear the heavier load, be it lived or dead bacilli [23]. Higher bacillary load was associated with poorer treatment outcome and higher mortality rate in tuberculosis [24]. Another possibility is treatment failure due to multidrug-resistant tuberculosis (MDR-TB) but we did not find many MDR-TB when we traced the sputum culture result. Higher sputum AFB load is something identifiable at diagnosis and reduction in bacillary load can be observed as early as three days with effective treatment [25]. It is perhaps worth considering an earlier and more rigorous follow up with sputum AFB smear for patients with higher bacillary load on diagnosis. The current practice in primary care clinics in Malaysia is to follow up after two to four weeks of treatment initiation, with sputum AFB smear being repeated then and after two months of intensive therapy. We would be able to detect poor response to treatment early and act accordingly.

In our study, we also included foreigners in our analysis. We found that foreigners in the state of Sabah were more likely to have delayed sputum conversion, similar to Mokti et al. [14]. The social demography in the state of Sabah is peculiar and different from the rest of Malaysia as they are known to have more foreigners. It has been estimated that non-citizen made up 30% of population in the state of Sabah, mainly from Indonesia and Philippines [26]. Foreign nationality was associated with poorer treatment outcome for tuberculosis in another local study [27]. It is not a practice among healthcare facilities to identify and deport any illegal immigrants who came to seek treatment for infectious diseases such as tuberculosis in Malaysia although they may be encouraged to continue treatment in their home countries. It is known that health-seeking behaviour is different among foreigners compared to locals, owing to various factors such as fear of deportation, financial constraints and language barriers [28]. Foreigners may tend to present late with higher severity of disease. That in turn may contribute to poorer treatment outcomes such as delayed sputum conversion. We also take note that this problem may be the tip of the iceberg as our study only captured foreigners who presented to the clinics, not those who avoided seeking medical treatment in government healthcare facilities.

We did not find a significant association with other variables that are significant in previous studies, namely the presence of diabetes mellitus, CXR severity, education level, smoking status, alcohol status, gap in treatment and duration of symptoms [2, 15, 19]. Of these factors, diabetes mellitus and smoking were more consistently captured in other studies as shown in Table 4 [14–16, 20, 21]. For diabetes mellitus, while many studies showed that its presence was associated with delayed sputum conversion, there were also studies which presented conflicting findings where presence of diabetes mellitus was not associated with poorer treatment outcome for TB such as delayed sputum conversion [29, 30]. Mahishale V (2017) reported a more specific finding where poor glycemic control upon diagnosis of TB predicted poorer outcomes such as delayed sputum conversion [31]. Shewade (2017) showed inadequate high-quality data in their systematic review to delineate the effect of glycaemic control on TB treatment outcome [32]. We hypothesized that our cohort of diabetic patients had better glycaemic control and thus, not significantly associated with delayed sputum conversion.

Smoking had also been associated with more extensive lung disease, lung cavitation, delayed sputum conversion at two months, higher default rates, treatment failures and relapses [33]. Ex-smokers also showed poorer treatment outcomes for tuberculosis [33]. The negative effect of smoking on TB treatment outcome would increase further if coupled with alcohol drinking [34]. Conflicting findings were also seen for smoking, where some studies found that smoking was not associated with negative outcome for tuberculosis [35, 36]. Bay JG (2022) posed an interesting hypothesis where they deduced that their cohort of smoker did not have poorer outcome due to better socioeconomic standing (employed, able to buy cigarettes, have better nutrition, better educated) [37]. In our study, we did not find any association of smoking with delayed sputum conversion. It may be possible that our cohort were mainly light or early smokers with no significant lung damage yet. More data would be needed in our cohort to explore the hypothesis of better socioeconomic profile as a protective factor against delayed sputum conversion.

An interesting finding for us is that the prolonged duration of symptoms before diagnosis of PTB was not significantly associated with delayed sputum conversion in our study. Some studies demonstrated that patients with longer duration of symptom more than 2–3 months had delayed conversion [38, 39]. This counter the argument that prolonged duration of symptoms may reflect higher mycobacterial burden due to progress of disease or prolonged exposure to source [2]. Objective measurement of disease burdens such as initial sputum AFB load and severity of CXR would be more useful in the initial assessment of patients with tuberculosis.

### Strength and limitation

Our study was a multicentre study where we included three main health clinics which were the treatment centre for tuberculosis, spanned across three different districts in Sabah. Another strength of our study was we extracted and verified data from patients’ medical records as well instead of just relying on the MyTB database. Certain variables were either not available or incomplete in MyTB database such as presence of other co-morbidities, alcohol status at diagnosis, duration of symptoms before diagnosis, sputum AFB load and number of days missing DOTS.

There were several limitations of our study. One, the sample size in our study may not be large enough to capture any significant difference. This was more evident for variables such as HIV status and presence of MDR-TB as their numbers were too small in our study for meaningful analysis. Two, our data on certain variables may not be that robust as well. While we did investigate the co-morbidities of our cohort, the co-morbidities varied a lot in the types of disease, rendering the numbers inadequate for analysis. A more detailed look into the characteristics of our patients with diabetes such as duration of disease, metabolic control including glycaemic, lipid and obesity profile, and smoking habits might be helpful to explain the insignificant results [40, 41]. Three, our outcome of sputum conversion was based on the result of sputum AFB smear. It was recognized that standard AFB smears could not differentiate dead bacilli from viable ones but performing routine sputum culture to demonstrate successful conversion was not feasible in resource-limited settings such as ours.

### Implications to practice and recommendations

Patients with risk factors for delayed sputum smear conversion at the completion of intensive phase of anti-tuberculous therapy (ATT) after two months should be identified earlier to avoid poorer disease outcomes, disease complications and possible progression to MDRTB. In this study, older age and high initial bacillary load at diagnosis were shown to be significantly associated with delayed sputum conversion. Patients with these identified risk factors should be properly and closely monitored and treated, with consideration of a more frequent follow up regime as opposed to current local practice, to ensure sputum conversion by the end of the intensive treatment phase.

It is also pertinent to recognize that most foreigners with symptoms are likely to present initially to a private primary healthcare provider. Malaysia health care system consists of a dual system in which the primary health care services are delivered by government health clinics and private general practices (GPs). In and around the capital city of the state of Sabah, Kota Kinabalu, there are approximately 300 GPs which act as a conduit for the initial recognition of and diagnosis of PTB in the population. The early identification of patients with risk factors for delayed sputum conversion, will help GPs to expedite the identification and referral of patients to an appropriate tertiary care centre for treatment to reduce the risk of spread of TB in the community.

Older person and foreigners are considered the vulnerable groups in this context. Future health policy should be designed to support these populations in terms of their follow up and treatment for tuberculosis to improve the rate of sputum conversion and ultimately treatment outcome for tuberculosis.

We also recommend that the MyTB database includes more robust data on clinical profile of patients such as the glycemic control upon diagnosis and the concurrent co-morbidities besides diabetes mellitus. The data would be useful for further research to elucidate the risk factors for poorer TB outcomes. Further research on the more effective follow up and treatment regime for patients at higher risk of delayed sputum conversion is recommended.

## Conclusion

The prevalence of delayed sputum conversion found in this study was considerably low at 8.8% (CI: 6.2–12.2). Foreigners, older person ≥ 60 years old and patients with high sputum AFB load (AFB 2+ and more) at diagnosis were at higher risk for delayed sputum conversion. Healthcare providers should take note of these factors and ensure the patients receive proper follow up treatment.

## Supporting information

S1 File Grading of pulmonary tuberculosis severity based on chest radiograph in adults. (DOCX)
