# Supplementary material for: Prevalence and associated factors of delayed sputum smear conversion in patients treated for smear positive pulmonary tuberculosis: A retrospective follow up study in Sabah, Malaysia
Source: PLoS One. 2023 Mar 6;18(3):e0282733. doi: 10.1371/journal.pone.0282733 (PMC9987811; doi:10.1371/journal.pone.0282733)
Supplement: S1 File — (DOCX) [file pone.0282733.s001.docx]

**Supporting information**

**Grading of Pulmonary Tuberculosis Severity Based on Chest Radiograph in Adults**

1. **Minimal**

Minimal lesions without demonstrable cavitations and confined to a small part of one or both lungs. The total extent of the lesions should not exceed the volume of the lung on one side which lies above the second chondrosternal junction and the spine of the fourth or the body of the fifth thoracic vertebrae.

1. **Moderate advanced**

One or both lungs may be involved but the total extent of the lesions should not exceed the following limits:

1. disseminated lesions of minimal to moderate density not exceeding the total volume of one lung or the equivalent in both lungs
2. dense and confluence lesions not exceeding one third of the volume of one lung
3. total diameter of cavitations, if present, must be <4 cm
4. **Far advanced**

Lesions are more extensive than moderately advanced

Source: Ministry of Health Malaysia. Clinical practice guidelines on management of tuberculosis. 4th ed. Putrajaya, Malaysia: Malaysian Health Technology Assessment Section; 2021.
